# Association between moderate to severe atopic dermatitis and lifestyle factors in the Dutch general population

**DOI:** 10.1111/ced.15212

**Published:** 2022-06-01

**Authors:** Junfen Zhang, Laura Loman, Marja Oldhoff, Marie L. A. Schuttelaar

**Affiliations:** ^1^ Department of Dermatology University of Groningen, University Medical Centre Groningen Groningen The Netherlands

## Abstract

**Background:**

Studies on the association between severity of atopic dermatitis (AD) and lifestyle factors in adults have not been conducted in the Netherlands to date.

**Aim:**

To explore the association between moderate to severe AD and lifestyle factors in adults in the Dutch general population.

**Methods:**

We conducted this cross‐sectional study within the Lifelines Cohort Study by sending a digital AD questionnaire to 135 950 adults in 2020. We extracted data on lifestyle factors from baseline, collected between 2006 and 2013. We analysed the association between lifestyle factors and presence of AD of any severity and of moderate to severe AD, using binary logistic regression and linear regression models.

**Results:**

We enrolled 56 896 participants (mean age 55.8 years, 39.7% males). The lifetime prevalence of self‐reported physician‐diagnosed AD was 9.1%, and the point prevalence of any AD and of moderate to severe AD was 3.3% and 2.3%, respectively. We found that moderate to severe AD was associated with smoking habit of > 15 pack‐years, alcohol consumption of > 2 drinks per day, chronic stress, Class I obesity, and both shorter and longer sleep duration. Moreover, we found dose–response associations with increases in smoking pack‐years and level of chronic stress. We observed no associations with abdominal obesity, physical activity, diet quality or a vegetarian/vegan diet.

**Conclusion:**

We found associations between moderate to severe AD and some modifiable lifestyle factors. Our findings indicate that more screening and counselling for lifestyle factors, particularly smoking, alcohol use, stress, obesity and sleep disturbances, appears warranted in patients with moderate to severe AD. Further longitudinal studies are required to better characterize the direction of these associations and to develop strategies for prevention.

## Introduction

Atopic dermatitis (AD) is a common disease, with a lifetime prevalence of self‐reported physician‐diagnosed AD of 7.1% among adults in the European general population.^1^ In the Dutch general population, 9.3% of adults have reported ever receiving a physician diagnosis of AD.^2^ AD is associated with worse health‐related quality of life (HRQoL), particularly in severely affected patients, and is associated with sleep disturbance and impaired mental health.^3^ Sleep disturbance may further contribute to impaired overall health in patients with AD.^4^

Unhealthy lifestyle factors such as smoking, obesity, alcohol use and a sedentary lifestyle, which may cause poor health outcomes, have been investigated in patients with AD, but contradictory results have been reported.^5^, ^6^, ^7^, ^8^, ^9^ Furthermore, the association between AD and certain lifestyle factors (e.g. smoking,^10^ obesity^11^, ^12^, ^13^ and alcohol use^10^) may correlate with AD severity, but the existing literature on this topic is limited. Understanding how the association with modifiable lifestyle factors varies depending on AD severity may help to monitor patients with moderate to severe AD in daily practice and to develop strategies for treatment and prevention. To date, studies on the association between moderate to severe AD and lifestyle factors among adults have not been conducted in the Netherlands.

The present study aimed to explore the association between moderate to severe AD and lifestyle factors among adults in the Dutch general population. The lifestyle factors studied included smoking, alcohol use, stress, obesity, physical activity, diet and sleep duration.

## Methods

### Study design and population

This cross‐sectional study was conducted by sending a digital add‐on questionnaire to adult participants in the Lifelines Cohort Study (*N* = 135 950) in 2020. The Lifelines Cohort Study^14^ is a multidisciplinary prospective population‐based cohort study, which is examining in a unique three‐generation design the health and health‐related behaviours of 169 729 individuals living in the north of the Netherlands. It uses a broad range of investigative procedures in assessing the biomedical, sociodemographic, behavioural, physical and psychological factors that contribute to the health and disease of the general population, with a special focus on multimorbidity and complex genetics.

Details of all questions used in this study, with category of outcomes and relevant references are shown in Supplementary Table S1. The add‐on questionnaire included questions related to AD and the occurrence of hand eczema (HE). Data on demographic factors, lifestyle factors and other diseases were extracted from baseline assessment, performed between 2006 and 2013.

### Atopic dermatitis definitions and severity assessment

Based on self‐reported physician‐diagnosed AD during lifetime, participants were categorized into AD and non‐AD. The point prevalence was determined as the proportion of the participants with physician‐diagnosed AD who reported current eczema in the past week. Regarding disease severity, participants with current eczema completed the Patient‐Oriented Eczema Measure (POEM),^15^ which asks seven questions on specific signs and symptoms of AD in the past week, with a total score of 0–28 and a severity banding of 0–7 (clear or mild) or 8–28 (moderate to severe).^15^

### Outcome measures of lifestyle factors

#### Smoking

Smoking status was defined as never, former and current, then current smoking was subclassified into > 0–7.9, ≥ 8–15 and > 15 cigarettes smoked per day. Smoking pack‐years in lifetime was classified into > 0–15 and > 15 pack‐years.

#### Alcohol consumption

Participants were classified into non‐, light, moderate and heavy drinkers according to their average daily alcohol consumption in the past month: 0, ≤ 1, > 1–2 and > 2 drinks per day, respectively.

#### Stress

The validated List of Threatening Events (LTE) and Long‐term Difficulties Inventory (LDI) were used for measuring stress in the past year, with higher scores indicating more stress.^16^ The LTE comprises 12 major categories of stressful life events (range sum score 0–12), whereas the LDI measures exposure to long‐term difficulties in 12 life domains (range sum score 0–24).^16^ The total scores were subdivided into categories: LTE 0, 1, 2 and ≥ 3; LDI: 0, 1–2, 3–4 and ≥ 5.

#### Obesity

Classifications of body mass index (BMI) and waist circumference (WC) were based on the WHO definition. Participants were divided into underweight, normal weight, overweight, Class I obesity and Class II/III obesity, based on their BMI: < 18.5, 18.5–24.9, 25–29.9, 30–34.9 and ≥ 35 kg/m^2^, respectively. Abdominal obesity was defined based on the WC: ≥ 102 cm for men and ≥ 88 cm for women.

#### Physical activity

The validated Short QUestionnaire to ASsess Health‐enhancing physical activity (SQUASH) was used to measure physical activity concerning a normal week in the past month.^17^ The SQUASH is prestructured into four domains: (i) commuting, (ii) leisure time and sports, (iii) household and (iv) occupational activities. Each domain assesses the duration and intensity of each activity.^17^ Intensity was based on a combination of self‐reported intensity and metabolic equivalent of tasks (METs).^18^

Physical activity was represented as the duration in min/week of moderate to vigorous physical activity (MVPA) and vigorous physical activity (VPA) performed. Based on their tertiles, duration of both was subclassified: MVPA 0, > 0–249, > 249–743 and > 743 min/week; VPA 0, > 0–120, > 120–295 and > 295 min/week.

#### Diet

Participants were dichotomized into vegetarian/vegan and nonvegetarian/nonvegan. Overall diet quality was assessed according to the Lifelines Diet Score (LLDS), which is based on the 2015 Dutch Dietary Guideline, with higher scores representing higher diet quality.^19^ It consists of 12 food groups: 9 food groups with proven positive health effects (vegetables, fruit, wholegrain products, legumes and nuts, fish, oils and soft margarine, unsweetened dairy, coffee and tea) and 3 food groups with negative effects (red and processed meat, butter and hard margarine, and sugar‐sweetened beverages). For each food group, the intake in grams per 1000 kilocalories (kcal) is categorized into quintiles, awarded 0–4 points (negative groups scored inversely) and summed (range sum score 0–48).^19^ Based on the LLDS quintiles, participants were divided into Q1 (0–18), Q2 (19–22), Q3 (23–25), Q4 (26–29) and Q5 (30–48).^19^

#### Sleep duration

Sleep duration in a 24‐h period was divided into ≤ 7, > 7–9 and > 9 h.

### Statistical analysis

Variables were analysed using descriptive statistics, including mean ± SD and proportions. The χ^2^ test was used to compare categorical variables in independent groups and the independent Student *t*‐test was used to compare continuous variables. Binary logistic regression and linear regression were performed to estimate odds ratios and β, respectively, and 95% CIs for the association between any AD or moderate to severe AD and lifestyle factors. Three adjusted models were constructed: Model 1 adjusted for age and sex; Model 2 adjusted for all potential confounders based on previous literature (i.e. age,^20^ sex,^20^ HE^21^ and asthma^22^, ^23^); and Model 3 adjusted for all potential confounders and all lifestyle factor variables (i.e. smoking status, alcohol, LDI, BMI, MVPA, vegetarian/vegan diet, LLDS and sleep duration). SPSS for Windows software (V25.0; IBM SPSS, Armonk, NY, USA) was used for all analyses. *P* < 0.05 was considered statistically significant.

## Results

### Study population

In total, 57 643 participants (42.4%) responded to the questions on AD, as described previously.^2^ After excluding 747 participants due to missing data on all lifestyle factors, 56 896 participants were included in our analysis (Table 1 summarizes the characteristics of the study population stratified for sex; Supplementary Data S2 shows the summary of missing values). The lifetime prevalence of self‐reported physician‐diagnosed AD was 9.1% (95% CI 8.9–9.4). The point prevalence of AD was 3.3% (95% CI 3.1–3.4) and the point prevalence of moderate to severe AD was 2.3% (95% CI 2.1–2.4).

**Table 1:** Characteristics of the study population from the lifelines cohort, stratified for sex.

Characteristic^a^	Total, *n* (%) (*N* = 56 896)	Male (*N* = 22 577)	Female (*N* = 34 319)	*P* ^b^
Age, years; mean ± SD	55.8 ± 12.2	57.3 ± 12.2	54.8 ± 12.1	**< 0.001**
Male	22 577 (39.7)	22 577 (100)	0	–
AD prevalence, *n* (%) [95% CI]				
Lifetime	5196 (9.1) [8.9–9.4]	1472 (6.5) [6.2–6.9]	3724 (10.9) [10.5–11.2]	**< 0.001**
Point	1840 (3.3) [3.1–3.4]	594 (2.7) [2.5–2.9]	1246 (3.7) [3.5–3.9]	**< 0.001**
Severity prevalence,^c^ *n* (%) [95% CI]				
Clear or mild	541 (1.0) [0.9–1.0]	205 (0.9) [0.8–1.0]	336 (1.0) [0.9–1.1]	0.36
Moderate to severe	1288 (2.3) [2.1–2.4]	386 (1.7) [1.5–1.9]	902 (2.7) [2.5–2.8]	**< 0.001**
Hand eczema, *n* (%) [95% CI]	8482 (15.0) [14.7–15.3]	2409 (10.7) [10.3–11.1]	6073 (17.8) [17.4–18.2]	**< 0.001**
Asthma, *n* (%) [95% CI]	4631 (8.2) [7.9–8.4]	1758 (7.8) [7.5–8.2]	2873 (8.4) [8.1–8.7]	**0.01**
Smoking				
Smoking status				**< 0.001**
Never smoker	26 253 (47.0)	9767 (43.9)	16 486 (48.9)	**< 0.001**
Former smoker	20 499 (36.7)	8509 (38.3)	11 990 (35.6)	**< 0.01**
Current smoker	9163 (16.4)	3953 (17.8)	5210 (15.5)	**< 0.001**
Cigarettes/day				
> 0–7.9	3622 (6.5)	1517 (6.8)	2105 (6.2)	**0.01**
8–15	3897 (7.0)	1612 (7.3)	2285 (6.8)	**0.03**
> 15	1644 (2.9)	824 (3.7)	820 (2.4)	**< 0.001**
Pack‐years				
0	26 252 (48.1)	9766 (45.3)	16 486 (50.0)	**< 0.001**
≤ 15	20 330 (37.3)	7591 (35.2)	12 739 (38.6)	**< 0.001**
> 15	7941 (14.6)	4198 (19.5)	3743 (11.4)	**< 0.001**
Alcohol consumption, drinks/day				
0 (nondrinker)	11 096 (21.0)	2187 (10.8)	8909 (27.3)	**< 0.001**
≤ 1 (light drinker)	26 534 (50.2)	8941 (44.3)	17 593 (54.0)	**< 0.001**
> 1–2 (moderate drinker)	11 152 (21.1)	6083 (30.1)	5069 (15.5)	**< 0.001**
> 2 (heavy drinker)	4025 (7.6)	2987 (14.8)	1038 (3.2)	**< 0.001**
Stress				
Total LTE score				
0	24 993 (44.7)	10 368 (46.8)	14 625 (43.3)	**< 0.001**
1	15 622 (27.9)	6124 (27.6)	9498 (28.1)	0.2
2	8881 (15.9)	3341 (15.1)	5540 (16.4)	**< 0.001**
≥ 3	6421 (11.5)	2325 (10.5)	4096 (12.1)	**< 0.001**
Total LDI score				
0	12 666 (22.7)	5802 (26.2)	6864 (20.3)	**< 0.001**
1–2	21 944 (39.3)	9240 (41.7)	12 704 (37.6)	**< 0.001**
3–4	12 492 (22.3)	4444 (20.1)	8048 (23.8)	**< 0.001**
≥ 5	8803 (15.7)	2671 (12.1)	6132 (18.2)	**< 0.001**
Obesity				
BMI, kg/m^2^				
Underweight (< 18.5)	397 (0.7)	59 (0.3)	338 (1.0)	**< 0.001**
Normal weight (18.5–24.9)	25 562 (44.9)	8526 (36.6)	17 306 (50.4)	**< 0.001**
Overweight (25–29.9)	22 663 (39.8)	11 157 (49.4)	11 506 (33.5)	**< 0.001**
Class I obesity (30–34.9)	6333 (11.1)	2605 (11.5)	3728 (10.9)	**0.01**
Class II/III obesity (≥ 35)	1922 (3.4)	492 (2.2)	1430 (4.2)	**< 0.001**
WC, cm				**< 0.001**
Male				
< 102	–	16 931 (75.0)	–	
≥ 102	–	5638 (25.0)	–	
Female				
< 88	–	–	20 255 (59.0)	
≥ 88	–	–	14 053 (41.0)	
Physical activity, min/week				
MVPA				
0	3345 (6.4)	1412 (6.9)	1933 (6.1)	**< 0.001**
> 0–249	16 463 (31.5)	5445 (26.6)	11 018 (34.6)	**< 0.001**
> 249–743	16 389 (31.3)	6008 (29.4)	10 381 (32.6)	**< 0.001**
> 743	16 122 (30.8)	7586 (37.1)	8536 (26.8)	**< 0.001**
VPA				
0	8094 (15.5)	3567 (17.4)	4527 (14.2)	**< 0.001**
> 0–120	15 829 (30.3)	5669 (27.7)	10 160 (31.9)	**< 0.001**
> 120–295	13 705 (26.2)	4899 (24.0)	8806 (27.6)	**< 0.001**
> 295	14 691 (28.1)	6316 (30.9)	8375 (26.3)	**< 0.001**
Diet				
Vegetarian/vegan	1183 (2.1)	251 (1.1)	932 (2.8)	**< 0.001**
Total LLDS score				
0–18	7549 (15.2)	3911 (19.9)	3638 (12.1)	**< 0.001**
19–22	10 317 (20.8)	4864 (24.7)	5453 (18.2)	**< 0.001**
23–25	9473 (19.1)	3957 (20.1)	5516 (18.4)	**< 0.001**
26–29	11 347 (22.8)	4075 (20.7)	7272 (24.2)	**< 0.001**
30–48	11 008 (22.2)	2884 (14.6)	8124 (27.1)	**< 0.001**
Sleep duration, h/day				
≤ 7	24 293 (43.3)	11 401 (51.3)	12 892 (38.0)	**< 0.001**
> 7–9	31 093 (55.4)	10 588 (47.7)	20 505 (60.4)	**< 0.001**
> 9	760 (1.4)	214 (1.0)	546 (1.6)	**< 0.001**

AD, atopic dermatitis; BMI, body mass index; LDI, Long‐term Difficulties Inventory; LLDS, Lifelines Diet Score; LTE, List of Threatening Events; MVPA, moderate to vigorous physical activity; VPA, vigorous physical activity; WC, waist circumference.

^a^ all characteristics excluding BMI and WC are self‐reported;

^b^ significant *P* values (< 0.05) are shown in bold;

^c^ severity measured according to the Patient‐Oriented Eczema Measure among the participants with physician‐diagnosed AD.

Nonresponders were younger, more often male and generally reported a less healthy lifestyle compared with responders (Supplementary Table S2).

### Potential confounders

In the univariate model, moderate to severe AD compared with non‐AD was positively associated with female sex, higher prevalence of HE and asthma, but inversely associated with age. These associations remained consistent in all multivariate models (Table 2).

**Table 2:** Association between moderate to severe atopic dermatitis and lifestyle factors using univariate and multivariate binary logistic regression analysis.

					Model 1^b^		Model 2^c^		Model 3^d^	
Binary logistic regression^a^	Non‐AD in lifetime, *n* (%) (*N* = 51 174)	Moderate to severe AD, *n* (%) (*N* = 1288)	Crude OR (95% CI^e^)	*P* ^e^	aOR (95% CI)	*P*	aOR (95% CI)	*P*	aOR (95% CI)	*P*
Age, years; mean ± SD	56.1 ± 12.1	51.1 ± 12.4	**0.97 (0.96–0.97)**	**< 0.001**	**0.97 (0.97–0.97)**	**< 0.001**	**0.98 (0.97–0.98)**	**< 0.001**	**0.98 (0.97–0.98)**	**< 0.001**
Sex										
Male	20 956 (41.0)	386 (30.0)	1	–	1	–	1	–	1	–
Female	30 218 (59.0)	902 (70.0)	**1.62 (1.44–1.83)**	**< 0.001**	**1.51 (1.34–1.70)**	**< 0.001**	**1.24 (1.09–1.40)**	**0.001**	**1.21 (1.04–1.41)**	**< 0.02**
Hand eczema										
No	45 172 (88.8)	530 (41.3)	1	–	1	–	1	–	1	–
Yes	5714 (11.2)	753 (58.7)	**11.23 (10.01–12.59)**	**< 0.001**	**10.35 (9.22–11.62)**	**< 0.001**	**10.05 (8.94–11.28)**	**< 0.001**	**9.61 (8.43–10.95)**	**< 0.001**
Asthma										
No	47 474 (93.0)	1011 (78.7)	1	–	1	–	1	–	1	–
Yes	3575 (7.0)	273 (21.3)	**3.59 (3.12–4.12)**	**< 0.001**	**3.28 (2.86–3.77)**	**< 0.001**	**2.95 (2.55–3.42)**	**< 0.001**	**3.03 (2.57–3.57)**	**< 0.001**
Smoking										
Smoking status				**< 0.001**		0.25		0.34		0.90
Never smoker	23 553 (46.8)	612 (49.2)	1	–	1	–	1	–	1	–
Former smoker	18 598 (37.0)	388 (31.2)	**0.80 (0.71–0.91)**	**0.001**	1.04 (0.91–1.19)	0.54	1.00 (0.87–1.15)	0.99	1.05 (0.90–1.23)	0.55
Current smoker	8144 (16.2)	245 (19.7)								
Cigarettes/day										
> 0–7.9	3219 (6.4)	94 (7.6)	1.12 (0.90–1.40)	0.30	1.10 (0.88–1.37)	0.55	1.15 (0.91–1.44)	0.24	1.11 (0.85–1.43)	0.45
8–15	3461 (6.9)	108 (8.7)	1.20 (0.98–1.48)	0.08	1.23 (1.00–1.51)	0.41	1.20 (0.97–1.49)	0.10	1.10 (0.86–1.41)	0.45
> 15	1464 (2.9)	43 (3.5)	1.13 (0.83–1.55)	0.44	1.25 (0.91–1.71)	0.05	1.17 (0.84–1.62)	0.35	1.07 (0.73–1.57	0.75
Pack‐years										
0	23 552 (48.0)	612 (50.6)	1	–	1	–	1	–	1	–
≤ 15	18 286 (37.3)	425 (35.2)	0.89 (0.79–1.01)	0.08	1.001 (0.89–1.15)	0.871	0.98 (0.86–1.12)	0.79	1.00 (0.86–1.16)	0.98
> 15	7189 (14.7)	172 (14.2)	0.92 (0.78–1.09)	0.34	**1.37 (1.14–1.64)**	**0.001**	**1.32 (1.09–1.59)**	**< 0.01**	**1.25 (1.01–1.54)**	**< 0.05**
Alcohol consumption, drinks/day				**0.001**		**0.01**		**< 0.01**		**0.01**
0 (nondrinker)	9849 (20.8)	267 (22.3)	1	–	1	–	1	–	1	–
≤ 1 (light drinker)	23 768 (50.1)	634 (53.0)	0.98 (0.85–1.14)	0.83	1.01 (0.87–1.17)	0.93	1.01 (0.87–1.18)	0.87	1.01 (0.85–1.21)	0.89
> 1–2 (moderate drinker)	10 160 (21.4)	196 (16.4)	**0.71 (0.59–0.86)**	**< 0.001**	0.87 (0.72–1.05)	0.14	0.88 (0.72–1.07)	0.20	0.86 (0.68–1.08)	0.18
> 2 (heavy drinker)	3655 (7.7)	99 (8.3)	1.00 (0.79–1.26)	0.99	**1.31 (1.02–1.67)**	**0.03**	**1.39 (1.08–1.79)**	**0.01**	**1.35 (1.01–1.79)**	**0.04**
Stress										
Total LTE score	–	–	–	**< 0.01**	–	**< 0.01**	–	0.21	–	0.29
0	22 684 (45.1)	511 (40.9)	1	–	1	–	1	–	1	–
1	13 963 (27.8)	356 (28.5)	1.13 (0.99–1.30)	0.08	1.14 (0.99–1.31)	0.07	1.08 (0.94–1.25)	0.27	1.06 (0.91–1.24)	0.46
2	7937 (15.8)	208 (16.6)	1.16 (0.99–1.37)	0.07	1.16 (0.99–1.37)	0.08	1.06 (0.89–1.26)	0.51	0.94 (0.77–1.14)	0.51
≥ 3	5698 (11.3)	175 (14.0)	**1.36 (1.15–1.62)**	**< 0.001**	**1.37 (1.15–1.63)**	**< 0.001**	**1.21 (1.01–1.46)**	**0.04**	1.17 (0.95–1.43)	0.14
Total LDI score	–	–	–	**< 0.001**	–	**< 0.001**	–	**< 0.001**	–	**< 0.001**
0	11 842 (23.6)	148 (11.8)	1		1		1		1	
1–2	19 897 (39.6)	447 (35.8)	**1.80 (1.49–2.17)**	**< 0.001**	**1.57 (1.30–1.90)**	**< 0.001**	**1.43 (1.18–1.74)**	**< 0.001**	**1.42 (1.15–1.75)**	**0.001**
3–4	11 045 (22.0)	331 (26.5)	**2.40 (1.97–2.92)**	**< 0.001**	**1.90 (1.56–2.32)**	**< 0.001**	**1.56 (1.27–1.92)**	**< 0.001**	**1.52 (1.21–1.90)**	**< 0.001**
≥ 5	7487 (14.9)	324 (25.9)	**3.46 (2.85–4.22)**	**< 0.001**	**2.53 (2.07–3.10)**	**< 0.001**	**2.00 (1.63–2.47)**	**< 0.001**	**1.92 (1.52–2.42)**	**< 0.001**
Obesity										
BMI, kg/m^2^				0.03		**0.01**		0.26		0.23
Underweight (< 18.5)	350 (0.7)	14 (1.1)	1.56 (0.91–2.68)	0.11	1.12 (0.65–1.93)	0.68	1.22 (0.69–2.14)	0.49	1.31 (0.73–2.37)	0.37
Normal weight (18.5–24.9)	22 946 (44.9)	588 (45.7)	1	–	1	–	1	–	1	–
Overweight (25–29.9)	20 491 (40.1)	472 (36.7)	0.90 (0.80–1.02)	0.09	1.12 (0.99–1.27)	0.08	1.05 (0.93–1.20)	0.44	1.03 (0.89–1.19)	0.71
Class I obesity (30–34.9)	5676 (11.1)	165 (12.8)	1.13 (0.95–1.35)	0.16	**1.38 (1.16–1.66)**	**< 0.001**	**1.23 (1.02–1.47)**	**0.03**	**1.25 (1.02–1.54)**	**0.03**
Class II/III obesity (≥ 35)	1696 (3.3)	48 (3.7)	1.10 (0.82–1.49)	0.51	1.19 (0.88–1.60)	0.27	0.97 (0.71–1.33)	0.84	0.98 (0.69–1.40)	0.91
WC, cm										
Male										
< 102	15 725 (75.1)	291 (75.6)	1	–	1	–	1	–	1	–
≥ 102	5226 (24.9)	94 (24.4)	0.97 (0.77–1.23)	0.81	1.15 (0.90–1.46)	0.27	1.06 (0.83–1.36)	0.64	1.04 (0.79–1.38)	0.78
Female										
< 88	17 758 (58.8)	540 (59.9)	1	–	1	–	1	–	1	–
≥ 88	12 450 (41.2)	362 (40.1)	0.96 (0.84–1.09)	0.52	1.14 (0.99–1.31)	0.06	1.03 (0.89–1.19)	0.74	1.03 (0.87–1.21)	0.74
Physical activity, min/week										
MVPA				**0.01**		0.22		0.26		0.09
0	2982 (6.3)	85 (7.3)	1	–	1	–	1	–	1	–
> 0–249	14 649 (31.1)	401 (34.6)	0.96 (0.76–1.22)	0.74	0.90 (0.7–1.15)	0.40	0.93 (0.72–1.19)	0.54	1.02 (0.78–1.33)	0.89
> 249–743	14 690 (31.2)	353 (30.4)	0.84 (0.66–1.07)	0.16	0.88 (0.69–1.12)	0.29	0.91 (0.71–1.17)	0.46	0.95 (0.72–1.25)	0.71
> 743	14 725 (31.3)	321 (27.7)	**0.77 (0.60–0.97)**	**0.03**	1.02 (0.80–1.31)	0.87	1.06 (0.82–1.36)	0.67	1.19 (0.90–1.56)	0.23
VPA				0.14		0.41		0.71	1	0.86
0	7306 (15.5)	195 (16.8)	1	–	1	–	1	–	1	–
> 0–120	14 172 (30.1)	374 (32.2)	0.99 (0.83–1.178)	1.00	0.94 (0.79–1.12)	0.46	0.95 (0.80–1.15)	0.62	1.01 (0.83–1.23)	0.95
> 120–295	12 313 (26.2)	290 (25.0)	0.88 (0.73–1.06)	0.18	0.86 (0.71–1.03)	0.10	0.90 (0.74–1.09)	0.27	0.97 (0.79–1.19)	0.75
> 295	13 255 (28.2)	301 (25.9)	0.85 (0.71–1.02)	0.08	0.91 (0.76–1.10)	0.34	0.97 (0.80–1.17)	0.73	1.05 (0.85–1.29)	0.66
Diet										
Vegetarian/vegan										
No	–	–	1	–	1	–	1	–	1	–
Yes	1043 (2.1)	32 (2.6)	1.24 (0.87–1.77)	0.23	1.151 (0.1–1.65)	0.44	0.89 (0.62–1.29)	0.55	1.14 (0.76–1.70)	0.53
Total LLDS score	–	–	–	0.07	–	0.98	–	0.84	–	0.90
0–18	6745 (15.1)	194 (17.3)	1	–	1	–	1	–	1	0.62
19–22	9275 (20.8)	250 (22.3)	0.94 (0.78–1.13)	0.50	1.03 (0.85–1.25)	0.76	1.04 (0.85–1.27)	0.69	1.06 (0.86–1.30)	0.61
23–25	8524 (19.1)	214 (19.1)	0.87 (0.72–1.06)	0.18	1.01 (0.83–1.24)	0.92	1.00 (0.81–1.23)	0.98	1.06 (0.85–1.32)	0.83
26–29	10 205 (22.8)	237 (21.2)	**0.82 (0.67–0.98)**	**0.03**	0.969 (0.80–1.18)	0.76	0.93 (0.76–1.15)	0.51	0.98 (0.79–1.22)	0.61
30–48	9936 (22.2)	224 (20.0)	**0.78 (0.65–0.95)**	**0.01**	0.97 (0.815–1.22)	0.92	1.01 (0.82–1.25)	0.93	1.06 (0.85–1.33)	0.43
Sleep duration, h/day	–	–	–	**0.03**	–	**0.02**	–	0.10	–	0.62
≤ 7	21 896 (43.4)	552 (43.3)	1.01 (0.90–1.13)	0.83	**1.15 (1.02–1.29)**	**0.02**	1.08 (0.96–1.22)	0.21	1.04 (0.90–1.18)	–
> 7–9	27 903 (55.3)	695 (54.5)	1	–	1	–	1	–	1	0.21
> 9	671 (1.3)	28 (2.2)	**1.68 (1.14–2.46)**	**< 0.01**	**1.48 (1.00–2.18)**	**< 0.05**	1.48 (0.98–2.23)	0.06	1.34 (0.85–2.11)	

AD, atopic dermatitis; BMI, body mass index; LDI, Long‐term Difficulties Inventory; LLDS, Lifelines Diet Score; LTE, List of Threatening Events; MVPA, moderate to vigorous physical activity; OR, adjusted odds ratio; VPA, vigorous physical activity; WC, waist circumference.

^a^ All characteristics excluding BMI and WC are self‐reported.

^b^ Model 1 included age and sex.

^c^ Model 2 included age, sex, asthma and hand eczema.

^d^ Model 3 included age, sex, asthma, hand eczema, smoking status, alcohol, LDI, BMI, MVPA, vegetarian/vegan, LLDS, sleep duration. Because of the overlap between variables, WC and sex, smoking status and smoking pack‐years, MVPA and VPA, and LTE and LDI were not entered at the same time in Model 3.

^e^ Bold type: if *P* < 0.05, it was considered statistically significant.

### Association between moderate to severe atopic dermatitis and lifestyle factors

The results from the binary logistic regression analysis regarding the association between moderate to severe AD and lifestyle factors are presented in Table 2, and linear regression analysis in Table 3 (see Supplementary Table S3 for the association between AD and lifestyle factors).

**Table 3:** Association between moderate to severe atopic dermatitis and lifestyle factors using univariate and multivariate linear regression analysis.

					Model 1^b^		Model 2^c^		Model^d^	
Characteristic^a^	Non‐AD in lifetime, mean ± SD	Moderate to severe AD, mean ± SD	Crude β (95% CI)	*P*	Adjusted β (95% CI)	*P*	Adjusted β (95% CI)	*P*	Adjusted β (95% CI)	*P*
Smoking pack‐years	6.0 ± 9.6	5.7 ± 9.3	0.97 (0.99–1.00)	0.27	1.01 (1.01–1.02)	< 0.001	1.01 (1.00–1.02)	< 0.01	1.01 (1.00–1.02)	0.03
No. of alcoholic drinks/day	0.8 ± 1.0	0.7 ± 1.0	0.94 (0.88–1.00)	0.05	1.04 (0.98–1.11)	0.19	1.06 (0.99–1.13)	0.11	1.05 (0.98–1.13)	0.21
Total LTE score	1.0 ± 1.2	1.1 ± 1.3	1.08 (1.04–1.13)	< 0.001	1.08 (1.04–1.13)	< 0.001	1.05 (1.00–1.09)	0.053	1.03 (0.98–1.09)	0.24
Total LDI score	2.3 ± 2.3	3.2 ± 2.6	1.15 (1.13–1.17)	< 0.001	1.11 (1.09–1.13)	< 0.001	1.08 (1.05–1.10)	< 0.001	1.07 (1.04–1.10)	< 0.001
BMI, kg/m^2^	25.9 ± 4.2	26.0 ± 4.5	1.00 (0.99–1.02)	0.82	1.02 (1.01–1.03)	0.001	1.01 (0.99–1.02)	0.29	1.01 (0.99–1.02)	0.49
WC, cm	90.0 ± 12.1	88.8 ± 12.7	0.99 (0.99–1.00)	0.001	1.01 (1.00–1.01)	< 0.01	1.00 (1.00–1.02)	0.48	1.00 (1.00–1.01)	0.64
LLDS score	24.8 ± 6.0	24.2 ± 6.0	0.98 (0.97–0.99)	< 0.01	1.0 (0.99–1.01)	0.58	1.00 (0.99–1.01)	0.65	1.00 (0.99–1.01)	0.94
Sleep duration, h/day	7.5 ± 0.9	7.5 ± 0.9	1.01 (0.95–1.08)	0.77	0.93 (0.87–0.99)	0.03	0.96 (0.90–1.03)	0.28	0.95 (0.88–1.03)	0.20

AD, atopic dermatitis; BMI, body mass index; LDI, Long‐term Difficulties Inventory; LTE, List of Threatening Events; MVPA, moderate to vigorous physical activity; VPA, vigorous physical activity; WC, waist circumference.

^a^ All characteristics excluding BMI and WC are self‐reported.

^b^ Model 1 included age and sex.

^c^ Model 2 included age, sex, asthma and hand eczema.

^d^ Model 3 included age, sex, asthma, hand eczema, smoking status, alcohol, LDI, BMI, MVPA, vegetarian/vegan, LLDS, sleep duration. Because of the overlap between variables, WC and sex, smoking status and smoking pack‐years, MVPA and VPA, and LTE and LDI were not entered at the same time in Model 3.

*P* < 0.05 is statistically significant.

In the univariate analysis, moderate to severe AD was positively associated with LTE score of ≥ 3, LDI score of > 0 for all categories and prolonged sleep duration of > 9 h/day, but inversely associated with former smoking, alcohol consumption of > 1–2 drinks/day, MVPA duration of > 743 min/week and high‐quality diet (LLDS 26–29 and LLDS 30–48).

These positive associations found in univariate analysis remained significant after adjusting for age and sex (Model 1). In addition, moderate to severe AD was positively associated with smoking habit of > 15 pack‐years, alcohol consumption of > 2 drinks/day, Class I obesity (BMI 30–34.9 kg/m^2^) and shorter sleep duration ≤ 7 h/day.

After controlling for all potential confounders (Model 2) and adjusting for all potential confounders and lifestyle factors (Model 3), the associations remained consistent between moderate to severe AD and LDI score of > 0 for all categories, smoking habit of > 15 pack‐years, alcohol consumption of > 2 drinks/day and Class I obesity (BMI 30–34.9 kg/m^2^).

Moreover, after replacing categorical variables with continuous variables, dose‐dependent associations between moderate to severe AD and increased smoking pack‐years and LDI score were found in all multivariate models.

## Discussion

In the present study, we found associations of moderate to severe AD with smoking habit of > 15 pack‐years, alcohol consumption of > 2 drinks per day, chronic stress, Class I obesity and altered sleep duration. Moreover, moderate to severe AD was associated with increased smoking pack‐years and level of chronic stress in a dose–response manner. No associations were observed with abdominal obesity, physical activity, diet quality or a vegetarian/vegan diet.

There are several potential mechanisms underlying the association between AD and lifestyle factors. Poor lifestyle factors may result in altered immune response, T helper (Th)1/Th2 imbalance and barrier dysfunction, thus contributing to the risk of AD.^24^, ^25^ Alternatively, AD, particularly moderate to severe AD, can impair the HRQoL of those affected, which may drive behaviour changes (e.g. increased smoking and alcohol use). Asthma and HE, common comorbidities related to AD, are also associated with certain lifestyle factors (e.g. smoking,^21^ obesity,^21^, ^22^ stress,^21^, ^23^ sleep disorders^21^). In the current study, after adjustment for the aforementioned factors, the associations of moderate to severe AD with higher smoking pack‐years, heavy alcohol use, chronic stress and Class I obesity remained consistent. Increased AD severity may also impact the association between AD and lifestyle factors, but existing epidemiological studies are limited.

In a meta‐analysis of 20 cross‐sectional studies, adult AD was associated with a higher prevalence of active smoking; however, the meta‐analysis did not further address the association between AD severity and smoking.^5^ A recent Danish nationwide register‐based study stratified smoking prevalence by AD severity and found an association of severe AD with smoking; in the study, AD severity was based on the use of systemic therapy and data on smoking were dichotomous.^10^ By contrast, we did not find an association of moderate to severe AD with former or current smoking in our study; this discrepancy may be due to differences in categorization of smoking, AD definitions and severity assessments. Furthermore, our study gives an insight into the association between AD severity and cumulative smoking dose, which has not previously been studied in the general population. We found an association between moderate to severe AD and a smoking habit of > 15 pack‐years, with a dose–response relationship. This finding may suggest a possible cumulative dose–response effect of tobacco smoking on disease severity.

A meta‐analysis of 13 studies found no association between AD prevalence and alcohol use.^7^ This meta‐analysis study also performed sub‐analyses by the amount of alcohol use and found no association between AD and heavy alcohol use whereas definitions of heavy alcohol use varied across studies.^7^ Of the included studies, only one cross‐sectional study from Denmark differentiated between mild and severe AD based on the use of systemic treatment and found more prevalent alcohol abuse among adults with AD compared with healthy controls, particularly among those with severe AD.^10^ The Danish study of alcohol abuse was defined by diagnoses or conditions strongly related to alcohol abuse.^10^ Our finding of an association between moderate to severe AD and heavy alcohol use of > 2 drinks per day is consistent with this Danish study, while we found no association with ≤ 1 or 1–2 alcoholic drinks per day. This may indicate a possible effect of high amounts of alcohol use on AD severity.

Our finding of an association between AD and chronic stress is consistent with previous studies.^23^, ^26^ A Danish population‐based study of 5648 adults showed that AD was associated with medium stress and high stress in a dose–response manner, where AD was defined by self‐reported allergic reactions and stress was based on self‐reported intensity and frequency of stress.^23^ Similarly, a Korean population‐based study of 33 018 adults found that adults with AD compared with healthy controls were more likely to rate a higher level of stress.^26^ Furthermore, an observational study reported an association between stress and both greater AD severity and increased scratching behaviour in patients with AD; in the study, the acute stress was induced in 15 patients with AD and 16 healthy participants, using the Trier Social Stress Test.^27^ This observational result may support the hypothesis that stress can trigger scratching behaviour and enhance the itch–scratch cycle in patients with AD, therefore aggravating disease severity.

A meta‐analysis of 30 studies found an association between adult AD and an increased prevalence of obesity; however, after pooling the European studies, the association did not remain significant.^6^ In this meta‐analysis, the association between AD severity and BMI was only addressed in children, where obesity was likely associated with increased disease severity.^6^ However, other recent studies^11^, ^12^, ^13^ found no association between obesity and increased AD severity in adults; however, AD definitions, severity assessments, BMI classification and methodology varied across these studies. A UK population‐based study of more than 2 million adults found no association between severe AD and being overweight or obese, but did find an association between mild or moderate AD and being overweight or obese.^11^ A US population‐based study of 8217 adults reported that moderate AD rather than severe AD was associated with obesity.^12^ Our finding of no association between moderate to severe AD and obesity in general is consistent with the findings of a large Israeli population‐based study,^13^ but after further stratifying by obesity, we did find an association between moderate to severe AD and Class I obesity, but not Class II obesity. This unusual pattern of the association may indicate that further studies, taking classes of obesity into account, will need to be performed.

Previous studies have shown conflicting results about whether AD is associated with decreased physical activity, despite a common belief that patients with AD may avoid exercise due to sweating and itch. In agreement with our finding, data from the Danish Skin Cohort showed that adults with moderate to severe AD reported similar levels of physical activity compared with healthy controls.^9^ A Swedish study of 110 adults with AD and 196 healthy participants found no significant differences in terms of the level of physical activity and their attitudes to physical performance.^28^ Conversely, data from the 2010 and 2012 National Health Interview Survey (NHIS) showed that adults with AD were less likely to perform VPA with lower frequency and duration.^29^ Another US population‐based study also found an association of AD with lower total counts of daily activity and MVPA, using actigraphy as an objective measure of physical activity.^8^ These inconsistent results may be attributable to differences in measures of physical activity and AD definitions and regional differences in attitudes towards fitness.

The association between adult AD and diet quality has, to the best of our knowledge, not previously been studied, although we found no association of moderate to severe AD with diet quality. The Phase 3 International Study of Asthma and Allergies in Childhood found similar results in children; childhood AD was not associated with diet quality based on Mediterranean diet score.^30^ Previous studies have indicated that antioxidant‐rich foods (e.g. vegetables and fruits) and polyunsaturated fatty acids (PUFAs) (found in foods such as margarine and fish) may have beneficial effects on AD by exerting antioxidant and immunomodulatory effects.^31^ The LLDS as an indicator of diet quality includes dietary information on various foods including antioxidant‐rich foods and PUFA; however, the current study did not identify an association of AD with LLDS.

We found that adults with moderate to severe AD were more likely to have both shorter and longer sleep duration. Shorter sleep duration is possibly caused by premature awakening and trouble falling asleep due to itch, whereas prolonged sleep duration may be caused by poor sleep quality, fatigue and daytime sleepiness. Moreover, greater AD severity may lead to more scratching behaviour and poorer sleep quality, which was supported by a small pilot study in which sleep efficacy of 20 adults with AD was assessed using objective measures (polysomnography and actigraphy).^32^ Notably, even though it is possible that the burden of sleep disturbance in patients with AD may severely affect overall health and increase healthcare utilization, sleep disorder was likely to be underdiagnosed and/or undertreated in patients with AD.^4^

The present study has several strengths: there was a large sample size, multiple lifestyle factors were assessed and validated self‐administered questionnaires were used to measure AD severity (POEM^15^), stress (LTE and LDI^16^), physical activity (SQUASH^17^) and diet quality (LLDS^19^). This study also has some limitations. This cross‐sectional study was unable to determine the direction of associations. Owing to the time interval between data collection on AD and lifestyle factors, participants might have changed some behaviours such as diet in the interval. Nonresponse bias is important to bear in mind, especially if caused by different sex ratios, because the sex of the patient has a bearing on both lifestyle factors (men are more likely to smoke, have a poorer diet, etc.) and the AD prevalence (female predilection). *P* values were not adjusted for multiple testing, although the *P* values of most of the significant associations were far smaller than 0.05.

## Conclusion

Associations between AD severity and some modifiable lifestyle factors were found in this study. Therefore, more screening and counselling for these lifestyle factors, particularly smoking, alcohol use, stress, obesity and sleep disturbances appear warranted in patients with moderate to severe AD. Further longitudinal studies are required to better characterize the direction of these associations and develop strategies for prevention.

**What's already known about this topic?**

- Previous studies provide evidence of the association between AD and sleep disturbances and stress.
- However, conflicting results concerning other lifestyle factors (e.g. smoking, obesity, alcohol use, a sedentary lifestyle) in patients with AD have been found.
- Associations with lifestyle factors (e.g. smoking, obesity and alcohol) may vary by disease severity of AD, but existing research on this topic is limited.

**What does this study add?**

- We found evidence of associations between moderate to severe AD and a lifetime smoking pack‐years of > 15, an alcohol consumption of > 2 drinks per day, chronic stress, Class I obesity and altered sleep duration among adults in the Dutch general population.
- Based on our results, we recommend more screening and counselling for lifestyle factors particularly smoking, alcohol use, stress, obesity and sleep disturbances in patients with moderate to severe AD.

## Conflict of interest

MLAS is an advisor, consultant, speaker and/or investigator for AbbVie, Pfizer, LEO Pharma, Regeneron, Sanofi Genzyme, Eli Lilly and Galderma, and has received grants from Regeneron, Sanofi Genzyme, Novartis and Pfizer. The other authors declare that they have no conflict of interest.

## Funding

This study was financially supported by Novartis; the funder had no role in the design or conduct of the study, the interpretation of the data, or the decision to submit the manuscript for publication. The Lifelines Biobank initiative has been made possible by subsidy from the Dutch Ministry of Health, Welfare and Sport, the Dutch Ministry of Economic Affairs, the University Medical Centre Groningen (UMCG the Netherlands), University Groningen and the Northern Provinces of the Netherlands. JZ was supported by the China Scholarship Council (CSC) Grant no. 201806200089.

## Ethics statement

All procedures were approved by the Medical Ethics Committee of University Medical Centre Groningen (reference numbers METc 2007/152 and METc 2019/571) and all participants provided written informed consent.

## Data availability

The data that support the findings of this study are from the Lifelines Cohort Study. Lifelines adheres to standards for open data availability and the data catalogue is publicly accessible on https://www.lifelines.nl/researcher/how‐to‐apply/catalogue. All international researchers can apply for data at the Lifelines research office. The Lifelines system allows access for reproducibility of the study results.

## Supporting information


**Data S1.** Summary of missing values.

**Table S1.** Questions and response options, and category of outcomes used in the current study, with relevant references.

**Table S2.** Nonresponder analysis.

**Table S3.** Association between physician‐diagnosed atopic dermatitis in lifetime and lifestyle factors using univariate and multivariate binary logistic regression and linear regression.

## References

[1] Svensson A , Ofenloch RF , Bruze M *et al*. Prevalence of skin disease in a population‐based sample of adults from five European countries. Br J Dermatol 2018; 178: 1111–18. 29247509 10.1111/bjd.16248

[2] Zhang J , Loman L , Voorberg AN *et al*. Prevalence of adult atopic dermatitis in the general population, with a focus on moderate‐to‐severe disease: results from the Lifelines Cohort Study. J Eur Acad Dermatol Venereol 2021; 35: e787–90. 34161629 10.1111/jdv.17471

[3] Drucker AM , Wang AR , Li W‐Q *et al*. The burden of atopic dermatitis: summary of a report for the National Eczema Association. J Invest Dermatol 2017; 137: 26–30. 27616422 10.1016/j.jid.2016.07.012

[4] Silverberg JI , Garg NK , Paller AS *et al*. Sleep disturbances in adults with eczema are associated with impaired overall health: a US population‐based study. J Invest Dermatol 2015; 135: 56–66. 25078665 10.1038/jid.2014.325

[5] Kantor R , Kim A , Thyssen JP *et al*. Association of atopic dermatitis with smoking: a systematic review and meta‐analysis. J Am Acad Dermatol 2016; 75: 1119–25.e1. 27542586 10.1016/j.jaad.2016.07.017 PMC5216172

[6] Zhang A , Silverberg JI . Association of atopic dermatitis with being overweight and obese: a systematic review and meta analysis. J Am Acad Dermatol 2015; 72: 606–16.e4. 25773409 10.1016/j.jaad.2014.12.013

[7] Halling‐Overgaard AS , Hamann CR , Holm RP *et al*. Atopic dermatitis and alcohol use – a meta‐analysis and systematic review. J Eur Acad Dermatol Venereol 2018; 32: 1238–45. 29377395 10.1111/jdv.14814

[8] Silverberg JI , Song J , Pinto D *et al*. Atopic dermatitis is associated with less physical activity in US adults. J Invest Dermatol 2016; 136: 1714–16. 27189827 10.1016/j.jid.2016.04.025 PMC5216175

[9] Egeberg A , Griffiths CEM , Williams HC *et al*. Clinical characteristics, symptoms and burden of psoriasis and atopic dermatitis in adults. Br J Dermatol 2020; 183: 128–38. 31630393 10.1111/bjd.18622

[10] Egeberg A , Andersen YM , Gislason GH *et al*. Prevalence of comorbidity and associated risk factors in adults with atopic dermatitis. Allergy 2017; 72: 783–91. 27864954 10.1111/all.13085

[11] Ascott A , Mansfield KE , Schonmann Y *et al*. Atopic eczema and obesity: a population‐based study. Br J Dermatol 2021; 184: 871–9. 33090454 10.1111/bjd.19597

[12] Silverberg JI , Gelfand JM , Margolis DJ *et al*. Association of atopic dermatitis with allergic, autoimmune, and cardiovascular comorbidities in US adults. Ann Allergy Asthma Immunol 2018; 121: 604–12.e3. 30092266 10.1016/j.anai.2018.07.042 PMC13217624

[13] Shalom G , Dreiher J , Kridin K *et al*. Atopic dermatitis and the metabolic syndrome: a cross‐sectional study of 116 816 patients. J Eur Acad Dermatology Venereol 2019; 33: 1762–7. 10.1111/jdv.15642 31045273

[14] Scholtens S , Smidt N , Swertz MA *et al*. Cohort profile: lifelines, a three‐generation cohort study and biobank. Int J Epidemiol 2015; 44: 1172–80. 25502107 10.1093/ije/dyu229

[15] Charman CR , Venn AJ , Ravenscroft JC *et al*. Translating patient‐oriented eczema measure (POEM) scores into clinical practice by suggesting severity strata derived using anchor‐based methods. Br J Dermatol 2013; 169: 1326–32. 24024631 10.1111/bjd.12590 PMC3920642

[16] Rosmalen JGM , Bos EH , De Jonge P . Validation of the Long‐term Difficulties Inventory (LDI) and the List of Threatening Experiences (LTE) as measures of stress in epidemiological population‐based cohort studies. Psychol Med 2012; 42: 2599–608. 22490940 10.1017/S0033291712000608

[17] Wendel‐Vos GC , Schuit AJ , Saris WH *et al*. Reproducibility and relative validity of the short questionnaire to assess health‐enhancing physical activity. J Clin Epidemiol 2003; 56: 1163–9. 14680666 10.1016/s0895-4356(03)00220-8

[18] Ainsworth BE , Haskell WL , Leon AS *et al*. Compendium of physical activities: classification of energy costs of human physical activities. Med Sci Sports Exerc 1993; 25: 71–80. 8292105 10.1249/00005768-199301000-00011

[19] Vinke PC , Corpeleijn E , Dekker LH *et al*. Development of the food‐based Lifelines Diet Score (LLDS) and its application in 129,369 Lifelines participants. Eur J Clin Nutr 2018; 72: 1111–19. 29895847 10.1038/s41430-018-0205-z

[20] Barbarot S , Auziere S , Gadkari A *et al*. Epidemiology of atopic dermatitis in adults: results from an international survey. Allergy 2018; 73: 1284–93. 29319189 10.1111/all.13401

[21] Loman L , Schuttelaar MLA . Hand eczema and lifestyle factors in the Dutch general population: evidence for smoking, chronic stress, and obesity. Contact Dermatitis 2022; 86: 80–8. 34766356 10.1111/cod.14005 PMC9300021

[22] Beuther DA , Sutherland ER . Overweight, obesity, and incident asthma: a meta‐analysis of prospective epidemiologic studies. Am J Respir Crit Care Med 2007; 175: 661–6. 17234901 10.1164/rccm.200611-1717OC PMC1899288

[23] Rod NH , Kristensen TS , Lange P *et al*. Perceived stress and risk of adult‐onset asthma and other atopic disorders: a longitudinal cohort study. Allergy 2012; 67: 1408–14. 22943607 10.1111/j.1398-9995.2012.02882.x.

[24] Hall JMF , Cruser D , Podawiltz A *et al*. Psychological stress and the cutaneous immune response: roles of the HPA axis and the sympathetic nervous system in atopic dermatitis and psoriasis. Dermatol Res Pract 2012; 2012: 403908. 22969795 10.1155/2012/403908 PMC3437281

[25] Han B , Wu WH , Bae JM *et al*. Serum leptin and adiponectin levels in atopic dermatitis (AD) and their relation to disease severity. J Am Acad Dermatol 2016; 75: 629–31. 27543217 10.1016/j.jaad.2016.04.036

[26] Park H , Kim K . Association of perceived stress with atopic dermatitis in adults: a population‐based study in Korea. Int J Env Res Public Health 2016; 13: 76. 10.3390/ijerph13080760 PMC4997446 27472355

[27] Mochizuki H , Lavery MJ , Nattkemper LA *et al*. Impact of acute stress on itch sensation and scratching behaviour in patients with atopic dermatitis and healthy controls. Br J Dermatol 2019; 180: 821–7. 29947106 10.1111/bjd.16921

[28] Lonne‐Rahm S‐B , Sundström I , Nordlind K *et al*. Adult atopic dermatitis patients and physical exercise: a Swedish questionnaire study. Acta Derm Venereol 2014; 94: 185–7. 23994911 10.2340/00015555-1556

[29] Silverberg JI , Greenland P . Eczema and cardiovascular risk factors in 2 US adult population studies. J Allergy Clin Immunol 2015; 135: 721–8.e6. 25579484 10.1016/j.jaci.2014.11.023

[30] Suárez‐Varela MM , Alvarez LG , Kogan MD *et al*. Diet and prevalence of atopic eczema in 6 to 7‐year‐old schoolchildren in Spain: ISAAC phase III. J Investig Allergol Clin Immunol 2010; 20: 469–75. 21243930

[31] Devereux G , Seaton A . Diet as a risk factor for atopy and asthma. J Allergy Clin Immunol 2005; 115: 1109–17. quiz 1118. 15940119 10.1016/j.jaci.2004.12.1139

[32] Bender BG , Ballard R , Canono B *et al*. Disease severity, scratching, and sleep quality in patients with atopic dermatitis. J Am Acad Dermatol 2008; 58: 415–20. 18280338 10.1016/j.jaad.2007.10.010

