# Supplementary material for: Association between moderate to severe atopic dermatitis and lifestyle factors in the Dutch general population
Source: Clin Exp Dermatol. 2022 Jun 1;47(8):1523–35. doi: 10.1111/ced.15212 (PMC9545670; doi:10.1111/ced.15212)
Supplement: Supplementary file 1 — Data S1. Summary of missing values. [file CED-47-1523-s001.docx]

# ****Supplementary material****

Moderate-to-severe atopic dermatitis and lifestyle factors in the Dutch general population

Junfen Zhang, Laura Loman,  [Jantje M. Oldhoff](https://pubmed.ncbi.nlm.nih.gov/?term=Oldhoff+JM&cauthor_id=33332048), Marie L.A. Schuttelaar

# Contents

| Table S1. Questions and response options, and category of outcomes used in the current study, with relevant references | 2 |
| --- | --- |
| Appendix S1 Summary of missing values | 8 |
| Table S2. Non-responder analysis | 9 |
| Table S3. Association between physician-diagnosed atopic dermatitis in lifetime and lifestyle factors using univariate and multivariate binary logistic regression and linear regression | 11 |
| References | 14 |

**Table S1.** Questions and response options, and category of outcomes used in the current study, with relevant references.

|  | **Questions and response options** | **Category of outcomes** | | **Reference** |
| --- | --- | --- | --- | --- |
| **Add-on questionnaire (collected in 2020)** | | | | |
| **Atopic dermatitis (AD)** | Have you ever been diagnosed with atopic dermatitis or atopic eczema by a physician?   - Yes - No | - physician-diagnosed AD  - non-AD in lifetime | Barbarot et al.,^1^ question P1 | |
| **Severity of atopic dermatitis** | Do you currently have eczema, which is present for at least 1 week?   - Yes, continue to the next question - No   Over the last week, on how many days has your skin been itchy because of your eczema?   - No days - 1-2 days - 3-4 days - 5-6 days - Every day   Over the last week, on how many nights has your sleep been disturbed because of your eczema?   - No days - 1-2 days - 3-4 days - 5-6 days - Every day   Over the last week, on how many days has your skin been bleeding because of your eczema?   - No days - 1-2 days - 3-4 days - 5-6 days - Every day   Over the last week, on how many days has your skin been weeping or oozing clear fluid because of your eczema?   - No days - 1-2 days - 3-4 days - 5-6 days - Every day   Over the last week, on how many days has your skin been cracked because of your eczema?   - No days - 1-2 days - 3-4 days - 5-6 days - Every day   Over the last week, on how many days has your skin been flaking off because of your eczema?   - No days - 1-2 days - 3-4 days - 5-6 days - Every day   Over the last week, on how many days has your skin felt dry or rough because of your eczema?   - No days - 1-2 days - 3-4 days - 5-6 days - Every day | Severity threshold scores on the Patient-Oriented Eczema Measure (POEM):  - 0-7 (clear or mild)  - 8-28 (moderate-to-severe) | Charman et al.,^2^ | |
| **Hand eczema (HE)** | Have you ever had hand eczema?   - Yes - No | - self-reported HE  - non HE in lifetime | Nordic Occupational Skin Questionnaire (NOSQ) 2002; question D1.^3^ | |
| **Baseline assessment of Lifelines (collected between 2006 and 2013)** | | | | |
| **Sex** | What is your sex?   - Male - Female | - male  - female | | None |
| **Age** | What is your year of birth? | - | | None |
| **Asthma** | Have you ever had asthma?   - Yes - No | - self-reported asthma  - non-asthma in lifetime | | None |
| **Skin diseases** | Could you indicate which of the following disorders you have (had)?  With several response options, including among others eczema, psoriasis, and severe acne. | - eczema  - psoriasis  - severe acne | | None |
| **Smoking** | **Smoking status**  (1) Have you ever smoked for as long as a year?   - Yes - No   (2) Do you smoke now or have you been smoking in the last month?   - Yes - No   (3) How many cigarettes/cigarillos/cigars/pipe tobacco do you smoke now on average per day? | Smoking status:  - never smoker (‘no’ to question 1)  - former smoker (‘yes’ to question 1 and ‘no’ to question 2)  - current smoker (‘yes’ to question 2)  According to the average number of cigarettes per day (question 3), current smoker was categorized into:  - >0-7.9  - 8-15  - >15  Note: When calculating the average number of cigarettes per day, both cigarillos and pipe tobacco were regarded as 1 cigarette, and cigars as 3 cigarettes. | | Meding et al.,^4^ |
|  | **Smoking pack years**  How much did you smoke up till now?  From age.. till age.. I smoked … cigarettes/cigarillos/cigars/pipe tobacco per day. | Smoking pack years was calculated as the number of packs of cigarettes (1 pack=20 cigarettes) smoked per day times the number of years of smoking, and was divided into:  - >0-15  - >15  Note: When calculating the average number of cigarettes per day, both cigarillos and pipe tobacco were regarded as 1 cigarette, and cigars as 3 cigarettes. | | None |
| **Alcohol consumption** | How often did you drink alcoholic drinks in the past month? (This includes non-alcoholic beer.)   - Not this month - 1 day per month - 2-3 days per month - 1 day per week - 2-3 days per week - 4-5 days per week - 6-7 days per week   How many glasses (of alcoholic drinks) did you drink on average on a drinking day?  With several response options, including 1, 2, 3, 4….11 and 12 or more. | Alcohol consumption was represented as the average number of alcoholic drinks per day, and was categorized into:  - 0 drink per day (non-drinker)  - ≤1 drink per day (light drinker)  - >1-2 drinks per day (moderate drinker)  - >2 drinks per day (heavy drinker) | | Slagter et al.,^5^ |
| **Stress** | **List of Threatening Events (LTE)**, a measure of acute stress, comprises 12 major categories of stressful life events. For each response category, participants indicate whether each of the 12 different life events occurred (yes/no) in the past year.   1. You were severely ill, severely injured, or a victim of violence 2. A relative was severely ill, severely injured, or a victim of violence 3. A parent, child, brother, sister, or partner died 4. A good friend or close relative died 5. You and your partner split up 6. You ended a long-term relationship with a good friend or relative 7. You got into a serious problem with a good friend, relative, or neighbour 8. You lost your job and haven't been able to find work again 9. You were fired 10. You faced severe financial difficulties 11. You got into trouble with the police or the law 12. You lost money or valuables, or these were stolen from you | The total LTE score ranges from 0 to 12, and was categorized into:  - 0  - 1  - 2  - ≥3 | | Rosmalen et al.,^6^ |
|  | **Long-term Difficulties Inventory (LDI)**, a measure of chronic stress, consists of 12 items referring to various aspects of life. For each response category, participants indicate how they experienced these aspects concerning difficulty and stress in the past year, on a three-point scale: 0=not stressful, 1= slightly stressful, 2=very stressful.   1. Home and living (e.g. accommodation too small, could not find a home, noise) 2. At or with work (e.g. too demanding, conflicts with boss, (imminent) dismissal) 3. Relationship with friends or acquaintances (e.g. quarrels, lack of support) 4. Relationship with your partner (e.g. jealousy, conflicts, doubt about the relationship, quarrels) 5. Relationship with your children (e.g. frequent conflicts, lack of respect for you) 6. Relationship with your parents (e.g. frequent conflicts, lack of acceptance) 7. Relationship with your other family members (e.g. frequent conflicts, lack of acceptance) 8. Free time (e.g. too little or too much free time) 9. Finances (e.g. major debts, insufficient income) 10. Your health (e.g. regularly ill, longer-term disorders) 11. School/study (too difficult, cannot be combined with other tasks) 12. Faith, church, or religion (e.g. doubt, conflicts with your minister) | The total LDI score ranges from 0 to 24, and was categorized into:  - 0  - 1-2  - 3-4  - ≥5 | | Rosmalen et al.,^6^ |
| **Obesity** | **Body Mass Index (BMI)**  Height and body weight without shoes and heavy clothing were measured at Lifelines research site, with the SECA 222 stadiometer and the SECA 761 scale.  BMI was calculated as weight divided by height squared (kilogram per square meter, kg/m^2^). | - underweight (BMI<18.5)  - normal weight (BMI 18.5-24.9)  - overweight (BMI 25-29.9)  - class I obesity (BMI 30-34.9)  - class II/III obesity (BMI≥35) | | World Health Organization (WHO) classification.^7^ |
|  | **Waist circumference (WC)**  The participant is requested to stand upright; SECA 201 measurement tape is placed between the lowest rib and the iliac crest around the bare stomach. Reading is at 0.5cm accurate. | The cut-off values for WC to define abdominal obesity were WC≥102 centimetre (cm) for males, and WC≥88cm for females. | | WHO classification.^8^ |
| **Physical activity** | The Short QUestionnaire to ASsess Health-enhancing physical activity (SQUASH) was used to evaluate habitual physical activities, regarding a normal week in recent months. The SQUASH is pre-structured into 4 domains: commuting, leisure-time and sports, household, and occupational activities, questions of which include 3 queries: days per week, average time per day, and intensity. The intensity of activities was evaluated based on the metabolic equivalent (MET) values from the Ainsworth Compendium of Physical Activities. | Based on its tertiles, moderate-to-vigorous physical activity (MVPA) duration in minutes per week was categorized into:  - 0  - >0-249  - >249-743  - >743  Based on its tertiles, vigorous physical activity (VPA) duration in minutes per week was categorized into:  - 0  - >0-120  - >120-295  - >295 | | Wendel-Vos et al.,^9^; Ainsworth et al.,^10^ |
| **Diet** | **Vegetarian/vegan**  (1) Were you on a diet based on certain beliefs/convictions (e.g. vegetarian or macrobiotic)?   - Yes, always - Yes, sometimes - No   (2) If you follow a diet based on certain beliefs/convictions, which belief/conviction is this?   - Vegetarian (meat is on the menu less than 1x per week) - Vegan (no animal products at all) - Macrobiotic - Anthroposophic - Belief/conviction: other | - vegetarian and/or vegan (‘yes, always’ to question 1 plus ‘vegetarian’ or ‘vegan’ to question 2)  -non-vegetarian/vegan | | None |
|  | **The Lifelines Diet Score (LLDS)**, a fully food-based and the 2015 Dutch Dietary Guidelines-based tool, was used to assess the overall diet quality, with higher scores representing a higher diet quality. It consists of 12 food groups, including 9 food groups with proven positive health effects (vegetables, fruit, whole grain products, legumes and nuts, fish, oil and soft margarine, unsweetened dairy, coffee, and tea) and 3 food groups with negative effects (red and processed meat, butter and hard margarine, and sugar-sweetened beverages). Per food group, the intake in grams per 1000 kilocalories is categorized into quintiles, awarded 0 to 4 points (negative groups scored inversely), and summed. The total LLDS ranges from 0 to 48. | According to its quintiles (Q), the total LLDS was categorized into:  - Q1 (0-18)  - Q2 (19-22)  - Q3 (23-25)  - Q4 (26-29)  - Q5 (30-48) | | Vinke et al.,^11^ |
| **Sleep duration** | On average how many hours do you sleep per full day (24 hours)?  On average how many minutes do you sleep per full day (24 hours)? | Sleep duration per full day was divided into:  - ≤7 hours  - >7-9 hours  - >9 hours | | None |

Abbreviation: AD, atopic dermatitis; POEM, Patient-Oriented Eczema Measure; HE, hand eczema; NOSQ, Nordic Occupational Skin Questionnaire; LTE, list of threatening events; LDI, long-term difficulties inventory; BMI, body mass index; kilogram per square meter, kg/m^2^; WHO, World Health Organization; WC, waist circumference; cm, centimetre; SQUASH, Short QUestionnaire to ASsess Health-enhancing physical activity; MET, metabolic equivalent; MVPA, moderate-to-vigorous physical activity; VPA, vigorous physical activity; LLDS, lifelines diet score; Q, quintile.

**Appendix S1.** Summary of missing values.

Table 1. Characteristics of the study population from the Lifelines cohort, stratified for sex.

Age: 0; lifetime prevalence: 149 males, 377 females; point prevalence: 132 males, 357 females; severity prevalence: 132 males, 362 females; hand eczema: 66 males, 142 females; asthma: 64 males, 82 females; smoking status: 348 males, 633 females; pack years: 1,022 males, 1,351 males; alcohol: 2,379 males, 1,710 females; LTE: 419 males, 560 females; LDI: 420 males, 571 females; BMI: 8 males, 11 females; WC: 8 males, 11 females; physical activity: 2,126 males, 2,451 females; vegetarian/vegan: 462 males, 646 females; LLDS: 2,886 males, 4,316 females; sleep duration: 374 males, 376 females.

Table 2. Association between moderate-to-severe atopic dermatitis and lifestyle factors using univariate and multivariate binary logistic regression and linear regression.

Non-AD in lifetime: age, sex: 0; hand eczema: 288; asthma: 125; smoking status: 879; pack years: 2,148; alcohol: 3,742; LTE: 892; LDI: 903; BMI: 15; WC: 5 males, 10 females; physical activity: 4,128; vegetarian/vegan: 1,005; LLDS: 6,489; sleep duration: 704. Moderate-to-severe AD: age, sex: 0; hand eczema: 5; asthma: 4; smoking status: 43; pack years: 79; alcohol: 92; LTE: 38; LDI: 38; BMI: 1; WC: 1 male, 0 female; physical activity: 128; vegetarian/vegan: 43; LLDS: 169; sleep duration: 13.

Table S2. Non-responder analysis.

Responders: age, sex : 0; asthma: 893; smoking status: 1,728; pack years: 3,120; alcohol: 4,836 ; LTE: 1,726; LDI: 1,738; BMI: 766; WC: 218 males, 548 females; physical activity: 5,324; vegetarian/vegan: 1,855; LLDS: 7,949; sleep duration: 1,497; Non-responders: age: 0; sex: 32; asthma: 448; smoking status: 3,586; pack years: 5,681; alcohol: 7,237; LTE: 3,814; LDI: 3,826; BMI: 24; WC: 10 males, 14 females; physical activity: 9,648; vegetarian/vegan: 3,968; LLDS: 10,757; sleep duration: 1,505.

Exact missing values regarding other skin diseases could not be calculated, as the relevant question was designed as a multiple response question, “Could you indicate which of the following disorders you have (had)?” with several response options, including among others eczema, psoriasis and severe acne. The missing values for the subjects who did not check any of the response options of this specific question were 2,128 responders, and 2,489 non-responder.

Table S3. Association between physician-diagnosed atopic dermatitis in lifetime and lifestyle factors using univariate and multivariate binary logistic regression and linear regression.

Non-AD: age, sex: 0; hand eczema: 288; asthma: 125; smoking status:879; pack years: 2,148; alcohol: 3,742; LTE: 892; LDI: 903; BMI: 15; WC: 5 males, 10 female; physical activity: 4,128; vegetarian/vegan: 1,005; LLDS: 6,489; sleep duration: 704. Physician-diagnosed AD: age, sex: 0; hand eczema: 53; asthma: 19; smoking status: 89; pack years: 200; alcohol: 311; LTE: 76; LDI: 76; BMI: 3; WC: 3 males, 0 female; physical activity: 397; vegetarian/vegan: 92; LLDS: 641; sleep duration: 40.

**Table S2.** Non-responder analysis.

|  | **Responders, n (%)**  **N=57,643** | **Non-responders, n (%)**  **N=77,147** | **p-value** |
| --- | --- | --- | --- |
| **Age,** years, mean ± SD | 55.3 ± 12.6 | 50.6 ± 12.3 | **<0.001** |
| **Sex**  Male  Female | 22,787 (39.5)  34,856 (60.5) | 32,993 (42,8)  44,122 (57.2) | **<0.001** |
| **Asthma** | 4,631 (8.2) | 7,219 (9.4) | **<0.001** |
| **Skin diseases**  Eczema  Psoriasis  Severe acne | 9,053 (15.7)  1,733 (3.0)  1,582 (2.7) | 11,592 (15.0)  1,995 (2.6)  2,254 (2.9) | **0.001**  **<0.001**  0.053 |
| **Smoking**  Smoking status  Never smoker  Former smoker  Current smoker, cig/day  >0-7.9  ≥8-15   >15  Pack years  0  ≤15  >15 | 26,253 (47.0)  20,499 (36.7)  3,622 (6.5)  3,897 (7.0)  1,644 (2.9)  26,252 (48.1)  20,330 (37.3)  7,941 (14.6) | 34,723 (47.2)  21,777 (29.6)  6,099 (8.3)  7,482 (10.2)  3,480 (4.7)  34,722 (48.6)  26,956 (37.7)  9,788 (13.7) | 0.369  **<0.001**  **<0.001**  **<0.001**  **<0.001**  0.124  0.117  **<0.001** |
| **Alcohol consumption**, drinks/day  0 (Non-drinker)  ≤1 (Light drinker)  >1-2 (Moderate drinker)  >2 (Heavy drinker) | 11,096 (21.0)  26,534 (50.2)  11,152 (21.1)  4,025 (7.6) | 13,944 (19.9)  35,764 (51.2)  14,237 (20.4)  5,970 (8.5) | **<0.001**  **0.002**  **0.001**  **<0.001** |
| **Stress**  Total LTE score  0  1  2  ≥3  Total LDI score  0  1-2  3-4  ≥5 | 24,993 (44.7)  15,622 (27.9)  8,881 (15.9)  6,421 (11.5)  12,666 (22.7)  21,944 (39.3)  12,492 (22.3)  8,803 (15.7) | 31,612 (43.1)  19,644 (26.8)  12,131 (16.5)  9,946 (13.6)  14,506 (19.8)  26,916 (36.7)  17,569 (24.0)  14,330 (19.5) | **<0.001**  **<0.001**  **0.001**  **<0.001**  **<0.001**  **<0.001**  **<0.001**  **<0.001** |
| **Obesity**  BMI, kg/m^2^  Underweight (<18.5)  Normal weight (18.5-24.9)  Overweight (25-29.9)  Class I obesity (30-34.9)  Class II/III obesity (≥35)  WC, cm  Male  <102  ≥102  Female  <88  ≥88 | 397 (0.7)  25,562 (44.9)  22,663 (39.8)  6,333 (11.1)  1,922 (3.4)  16,931 (75.0)  5,638 (25.0)  20,255 (59.0)  14,053 (41.0) | 647 (0.8)  34,828 (45.2)  29,739 (38.6)  8,988 (11.7)  2,921 (3.8)  25,332 (76.8)  7,651 (23.2)  26,323 (59.7)  17,785 (40.3) | **0.004**  0.431  **<0.001**  **0.003**  **<0.001**  **<0.001**  <0.070 |
| **Physical activity**, min/wk  MVPA  0  >0-249  >249-743  >743  VPA  0  >0-120  >120-295  >295 | 3,345 (6.4)  16,463 (31.5)  16,389 (31.3)  16,122 (30.8)  8,094 (15.5)  15,829 (30.3)  13,705 (26.2)  14,691 (28.1) | 6,153 (9.1)  23,289 (34.5)  20,295 (30.1)  17,762 (26.3)  13,005 (19.3)  20,958 (31.0)  16,654 (24.7)  16,882 (25.0) | **<0.001**  **<0.001**  **<0.001**  **<0.001**  **<0.001**  **0.003**  **<0.001**  **<0.001** |
| **Diet**  Vegetarian/Vegan  Total LLDS score  0-18  19-22  23-25  26-29  30-48 | 1,183 (2.1)  7,549 (15.2)  10,317 (20.8)  9,473 (19.1)  11,347 (22.8)  11,008 (22.2) | 1,279 (1.7)  14,272 (21.5)  15,845 (23.9)  12,514 (18.8)  13,174 (19.8)  10,585 (15.9) | **<0.001**  **<0.001**  **<0.001**  0.358  **<0.001**  **<0.001** |
| **Sleep duration**, h/day  ≤7  >7-9  > 9 | 24,293 (43.3)  31,093 (55.4)  760 (1.4) | 33,537 (44.6)  40,406 (53.7)  1,299 (1.7) | **<0.001**  **<0.001**  **<0.001** |

All characteristics excluding BMI and WC are self-reported.

Abbreviation: n, number; SD, standard deviation; cig, cigarettes; LTE, list of threatening events; LDI, long-term difficulties inventory; BMI, body mass index; kg/m^2^, kilogram per square meter; WC, waist circumference; cm, centimetre; min, minutes; wk, week; MVPA, moderate-to-vigorous physical activity; VPA, vigorous physical activity; LLDS, lifelines diet score; h, hour.

**Table S3.** Association between physician-diagnosed atopic dermatitis in lifetime and lifestyle factors using univariate and multivariate binary logistic regression and linear regression.

| Logistic regression | **Non-AD, n (%)**  **N=51,174** | **AD, n (%)**  **(N=5,196)** | **Crude OR (95% CI)** | **P-value** | **aOR (95% CI)**  **Model 1** | **p-value** | **aOR (95% CI)**  **Model 2** | **p-value** | **aOR (95% CI)**  **Model 3** | **p-value** |
| --- | --- | --- | --- | --- | --- | --- | --- | --- | --- | --- |
| **Age, years, mean ± SD** | 56.1 ± 12.1 | 52.5 ± 11.9 | **0.976 (0.974-0.978)** | **<0.001** | **0.978 (0.975-0.980)** | **<0.001** | **0.983 (0.980-0.985)** | **<0.001** | **0.984 (0.981-0.987)** | **<0.001** |
| **Sex**  Male  Female | 20,956 (41.0)  30,218 (59.0) | 1,472 (28.3)  3,724 (71.7) | 1  **1.754 (1.648-1.868)** | **<0.001** | 1  **1.663 (1.561-1.772)** | **<0.001** | 1  **1.443 (1.350-1.543)** | **<0.001** | 1  **1.424 (1.313-1.544)** | **<0.001** |
| **Hand eczema**  No  Yes | 45,172 (88.6)  5,714 (11.2) | 2,729 (52.7)  2,414 (46.6) | 1  **6.993 (6.577-7.435)** | **<0.001** | 1  **6.481 (6.092-6.895)** | **<0.001** | 1  **6.342 (5.957-6.751)** | **<0.001** | 1  **6.053 (5.645-6.492)** | **<0.001** |
| **Asthma**  No  Yes | 47,474 (93.0)  3,575 (7.0) | 4,217 (81.5)  960 (18.5) | 1  **3.023 (2.797-3.268)** | **<0.001** | 1  **2.839 (2.624-3.073)** | **<0.001** | 1  **2.637 (2.422-2.870)** | **<0.001** | 1  **2.635 (2.397-2.896)** | **<0.001** |
| **Smoking**  Smoking status  Never smoker  Former smoker  Current smoker, cig/day  **>**0-7.9  ≥8-15  >15  Pack years  0  ≤15  >15 | 23,553 (46.8)  18,598 (37.0)  3,219 (6.4)  3,461 (6.9)  1,464 (2.9)  23,552 (48.0)  18,286 (37.3)  7,189 (14.7) | 2,476 (48.5)  1,718 (33.6)  361 (7.1)  391 (7.7)  161 (3.2)  2,476 (49.6)  1,842 (36.9)  678 (13.6) | 1  **0.879 (0.824-0.937)**  1.067 (0.950-1.198)  1.075 (0.960-1.203)  1.046 (0.884-1.237)  1  0.958 (0.899-1.021)  **0.897 (0.821-0.981)** | **<0.001**  **<0.001**  0.276  0.210  0.599  **0.047**  0.186  **0.017** | 1  **1.073 (1.002-1.148)**  1.056 (0.939-1.188)  1.105 (0.987-1.238)  1.151 (0.972-1.363)  1  1.045 (0.980-1.115)  **1.219 (1.111-1.339)** | 0.114  **0.043**  0.359  0.083  0.104  **<0.001**  0.178  **<0.001** | 1  1.047 (0.974-1.125)  1.108 (0.979-1.254)  1.074 (0.953-1.211)  1.083 (0.905-1.297)  1  1.031 (0.963-1.104)  **1.178 (1.067-1.300)** | 0.350  0.210  0.105  0.242  0.383  **0.005**  0.376  **0.001** | 1  1.029 (0.949-1.116)  1.020 (0.886-1.174)  0.987 (0.859-1.133)  1.015 (0.822-1.253)  1  0.997 (0.923-1.077)  1.107 (0.988-1.239) | 0.958  0.485  0.781  0.850  0.889  0.162  0.937  0.079 |
| **Alcohol consumption**, drinks/day  0 (Non-drinker)  ≤1 (Light drinker)  >1-2 (Moderate drinker)  >2 (Heavy drinker) | 9,849 (20.8)  23,768 (50.1)  10,160 (21.4)  3,655 (7.7) | 1,137 (23.3)  2,512 (51.4)  904 (18.5)  332 (6.8) | 1  **0.916 (0.850-0.986)**  **0.771 (0.703-0.845)**  **0.787 (0.692-0.894)** | <0.001  **0.019**  **<0.001**  **<0.001** | 1  0.959 (0.890-1.033)  0.955 (0.868-1.049)  1.077 (0.942-1.232) | 0.204  0.269  0.335  0.275 | 1  0.968 (0.894-1.048)  0.970 (0.877-1.072)  1.136 (0.986-1.308) | 0.098  0.424  0.550  0.077 | 1  0.966 (0.880-1.061)  0.972 (0.866-1.092)  1.143 (0.977-1.337) | 0.108  0.472  0.634  0.094 |
| **Stress**  Total LTE score  0  1  2  ≥3  Total LDI score  0  1-2  3-4  ≥5 | 22,684 (45.1)  13,963 (27.8)  7,937 (15.8)  5,698 (11.3)  11,842 (23.6)  19,897 (39.6)  11,045 (22.0)  7,487 (14.9) | 2,114 (41.3)  1,503 (29.4)  843 (16.5)  660 (12.9)  736 (14.4)  1,866 (36.4)  1,318 (25.7)  1,200 (23.4) | 1  **1.155 (1.078-1.238)**  **1.140 (1.048-1.239)**  **1.243 (1.134-1.363)**  1  **1.509 (1.381-1.648)**  **1.920 (1.748-2.109)**  **2.579 (2.342-2.839)** | **<0.001**  **<0.001**  **0.002**  **<0.001**  **<0.001**  **<0.001**  **<0.001**  **<0.001** | 1  **1.154 (1.076-1.237)**  **1.130 (1.039-1.230)**  **1.231 (1.122-1.351)**  1  **1.365 (1.248-1.492)**  **1.600 (1.454-1.762)**  **2.013 (1.822-2.225)** | **<0.001**  **<0.001**  **0.004**  **<0.001**  **<0.001**  **<0.001**  **<0.001**  **<0.001** | 1  **1.107 (1.028-1.192)**  1.060 (0.969-1.159)  **1.130 (1.024-1.248)**  1  **1.281 (1.167-1.406)**  **1.411 (1.276-1.561)**  **1.719 (1.548-1.910)** | **0.017**  **0.007**  0.202  **0.015**  **<0.001**  **<0.001**  **<0.001**  **<0.001** | 1  **1.124 (1.036-1.219)**  1.041 (0.942-1.150)  **1.145 (1.026-1.279)**  1  **1.281 (1.155-1.421)**  **1.458 (1.303-1.630)**  **1.780 (1.583-2.001)** | **0.013**  **0.005**  0.433  **0.016**  **<0.001**  **<0.001**  **<0.001**  **<0.001** |
| **Obesity**  BMI, kg/m^2^  Underweight (<18.5)  Normal weight (18.5-24.9)  Overweight (25-29.9)  Class I obesity (30-34.9)  Class II/III obesity (≥35)  WC, cm  Male  <102  ≥102  Female  <88  ≥88 | 350 (0.7)  22,946 (44.9)  20,491 (40.1)  5,676 (11.1)  1,696 (3.3)  15,725 (75.1)  5,226 (24.9)  17,758 (58.8)  12,450 (41.2) | 44 (0.8)  2,411 (46.4)  1,942 (37.4)  600 (11.6)  196 (3.8)  1,103 (75.1)  366 (24.9)  2,286 (61.4)  1,438 (38.6) | 1.196 (0.872-1.642)  1  **0.902 (0.847-0.960)**  1.006 (0.916-1.105)  1.100 (0.943-1.283)  1  0.998 (0.884-1.128)  1  **0.897 (0.837-0.962)** | **0.002**  0.266  **0.001**  0.900  0.225  0.980  **0.002** | 0.915 (0.665-1.258)  1  **1.090 (1.022-1.163)**  **1.173 (1.066-1.290)**  1.135 (0.973-1.325)  1  1.087 (0.960-1.231)  1  1.025 (0.954-1.102) | **0.005**  0.584  **0.009**  **0.001**  0.108  0.189  0.496 | 0.948 (0.678-1.325)  1  1.052 (0.983-1.126)  1.068 (0.965-1.182)  0.998 (0.846-1.178)  1  1.029 (0.903-1.172)  1  0.962 (0.891-1.039) | 0.529  0.755  0.144  0.205  0.982  0.672  0.321 | 0.928 (0.644-1.337)  1  1.038 (0.962-1.120)  1.040 (0.928-1.166)  1.046 (0.870-1.256)  1  0.989 (0.852-1.147)  1  0.943 (0.865-1.028) | 0.847  0.689  0.339  0.497  0.634  0.879  0.183 |
| **Physical activity**, min/wk  MVPA  0  >0-249  >249-743  >743  VPA  0  >0-120  >120-295  >295 | 2,982 (6.3)  14,649 (31.1)  14,690 (31.2)  14,725 (31.3)  7,306 (15.5)  14,172 (30.1)  12,313 (26.2)  13,255 (28.2) | 324 (6.8)  1,671 (34.8)  1,546 (32.2)  1,258 (26.2)  709 (14.8)  1,514 (31.5)  1,265 (26.4)  1,311 (27.3) | 1  1.050 (0.926-1.190)  0.969 (0.854-1.099)  **0.786 (0.692-0.894)**  1  **1.101 (1.003-1.209)**  1.059 (0.961-1.166)  1.019 (0.926-1.122) | **<0.001**  0.447  0.620  **<0.001**  0.126  **0.044**  0.246  0.697 | 1  0.992 (0.875-1.126)  0.995 (0.877-1.130)  0.982 (0.862-1.119)  1  1.043 (0.949-1.146)  1.025 (0.930-1.129)  1.082 (0982-1.191) | 0.988  0.906  0.942  0.791  0.389  0.383  0.621  0.111 | 1  1.022 (0.894-1.169)  1.020 (0.891-1.167)  1.007 (0.877-1.157)  1  1.079 (0.976-1.192)  1.086 (0.980-1.204)  **1.136 (1.025-1.258)** | 0.977  0.746  0.775  0.920  0.113  0.137  0.114  **0.015** | 1  1.050 (0.909-1.213)  1.027 (0.887-1.189)  1.031 (0.887-1.198)  1  1.112 (0.998-1.238)  **1.121 (1.002-1.254)**  **1.165 (1.041-1.304)** | 0.901  0.507  0.722  0.690  0.067  0.054  **0.046**  **0.008** |
| **Diet**  Vegetarian/Vegan  No  Yes  Total LLDS score  0-18  19-22  23-25  26-29  30-48 | 49,126 (97.9)  1,043 (2.1)  6,745 (15.1)  9,275 (20.8)  8,524 (19.1)  10,205 (22.8)  9,936 (22.2) | 4,976 (97.5)  128 (2.5)  731 (16.0)  948 (20.8)  870 (19.1)  1,021 (22.4)  985 (21.6) | 1  **1.212 (1.006-1.459)**  1  0.943 (0.852-1.044)  0.942 (0.849-1.044)  0.923 (0.835-1.020)  0.915 (0.827-1.011) | **0.043**  0.477  0.258  0.255  0.116  0.082 | 1  1.110 (0.921-1.339)  1  1.002 (0.904-1.111)  1.028 (0.925-1.142)  1.024 (0.923-1.135)  1.033 (0.929-1.150) | 0.274  0.958  0.965  0.609  0.654  0.545 | 1  1.094 (0.898-1.333)  1  0.993 (0.891-1.107)  1.013 (0.906-1.133)  0.995 (0.892-1.110)  1.034 (0.924-1.157) | 0.373  0.934  0.901  0.820  0.932  0.558 | 1  1.038 (0.836-1.289)  1  0.998 (0.891-1.119)  1.049 (0.933-1.180)  1.023 (0.911-1.148)  1.059 (0.939-1.194) | 0.734  0.787  0.979  0.422  0.702  0.351 |
| **Sleep duration**, h/day  ≤7  >7-9  >9 | 21,896 (43.4)  27,903 (55.3)  671 (1.3) | 2,183 (42.3)  2,895 (56.1)  78 (1.5) | 0.961 (0.907-1.019)  1  1.120 (0.883-1.421) | 0.226  0.180  0.348 | **1.073 (1.011-1.139)**  1  1.015 (0.799-1.290) | 0.066  **0.020**  0.903 | 1.026 (0.963-1.092)  1  0.983 (0.761-1.271) | 0.714  0.427  0.898 | 0.983 (0.917-1.055)  1  0.879 (0.662-1.166) | 0.624  0.643  0.370 |
| Linear regression | **Non-AD, mean ± SD** | **AD in lifetime**  **mean ± SD** | **Crude β (95% CI)** | **P-value** | **adjusted β (95% CI)**  **Model 1** | **p-value** | **adjusted β (95% CI)**  **Model 2** | **p-value** | **adjusted β (95% CI)**  **Model 3** | **p-value** |
| **Smoking pack years** | 6.0 ± 9.6 | 5.6 ± 9.1 | **0.996 (0.993-0.999)** | **0.007** | **1.008 (1.004-1.011)** | **<0.001** | **1.007 (1.003-1.010)** | **<0.001** | **1.005 (1.001-1.009)** | **0.020** |
| **No. of alcoholic drinks/day** | 0.8 ± 1.0 | 0.7 ± 0.9 | **0.906 (0.877-0.937)** | **<0.001** | 1.013 (0.979-1.048) | 0.451 | 1.023 (0.987-1.060) | 0.211 | 1.016 (0.977-1.057) | 0.414 |
| **Total LTE score** | 1.0 ± 1.2 | 1.1 ± 1.3 | **1.063 (1.040-1.087)** | **<0.001** | **1.060 (1.037-1.084)** | **<0.001** | **1.033 (1.009-1.058)** | **0.007** | **1.037 (1.010-1.065)** | **0.008** |
| **Total LDI score** | 2.3 ± 2.3 | 3.0 ± 2.5 | **1.121 (1.109-1.134)** | **<0.001** | **1.087 (1.074-1.099)** | **<0.001** | **1.064 (1.051-1.077)** | **<0.001** | **1.067 (1.052-1.082)** | **<0.001** |
| **BMI, kg/m^2^** | 25.9 ± 4.2 | 25.9 ± 4.4 | 0.996 (0.989-1.003) | 0.270 | **1.012 (1.005-1.018)** | **0.001** | 1.003 (0.995-1.010) | 0.486 | 1.001 (0.993-1.009) | 0.766 |
| **WC, cm** | 90.0 ± 12.1 | 88.4 ± 12.5 | **0.989 (0.987-0.991)** | **<0.001** | **1.003 (1.000-1.005)** | **0.038** | 0.999 (0.996-1.002) | 0.496 | 0.998 (0.995-1.001) | 0.223 |
| **LLDS score** | 24.8 ± 6.0 | 24.6 ± 6.0 | **0.995 (0.990-1.000)** | **0.057** | 1.002 (0.996-1.007) | 0.527 | 1.002 (0.996-1.008) | 0.529 | 1.003 (0.997-1.009) | 0.346 |
| **Sleep duration, h/day** | 7.5 ± 0.9 | 7.5 ± 0.9 | 1.030 (0.996-1.064) | 0.081 | **0.958 (0.926-0.991)** | **0.013** | 0.985 (0.951-1.021) | 0.420 | 0.994 (0.955-1.034) | 0.769 |

All characteristics excluding BMI and WC are self-reported.

Abbreviation: AD, atopic dermatitis; OR, odds ratio; CI, confidence interval; aOR, adjusted odds ratio; SD, standard deviation; cig, cigarettes; LTE, list of threatening events; LDI, long-term difficulties inventory; BMI, body mass index; kg/m^2^, kilogram per square meter; WC, waist circumference; cm, centimetre; min, minutes; wk, week; MVPA, moderate-to-vigorous physical activity; VPA, vigorous physical activity; LLDS, lifelines diet score; h, hour; no., number.

Model-1 included age, sex; Model-2 included age, sex, asthma, hand eczema; Model-3 included age, sex, asthma, hand eczema, smoking status, alcohol, LDI, BMI, MVPA, vegetarian/vegan, LLDS, sleep duration.

Considering the overlap between variables, WC and sex, smoking status and smoking pack years, MVPA and VPA, LTE and LDI, were not entered at the same time in model 3.

**References**

1. Barbarot S, Auziere S, Gadkari A, et al. Epidemiology of atopic dermatitis in adults: Results from an international survey. *Allergy* 2018; 73: 1284–1293.

2. Charman CR, Venn AJ, Ravenscroft JC, et al. Translating patient-oriented eczema measure (POEM) scores into clinical practice by suggesting severity strata derived using anchor-based methods. *Br J Dermatol* 2013; 169: 1326–1332.

3. Susitaival P, Flyvholm MA, Meding B, et al. Nordic Occupational Skin Questionnaire (NOSQ-2002): A new tool for surveying occupational skin diseases and exposure. *Contact Dermatitis* 2003; 49: 70–76.

4. Meding B, Alderling M, Wrangsjö K. Tobacco smoking and hand eczema: a population-based study. *Br J Dermatol* 2010; 163: 752–756.

5. Slagter SN, van Vliet-Ostaptchouk J V, Vonk JM, et al. Combined effects of smoking and alcohol on metabolic syndrome: the LifeLines cohort study. *PLoS One* 2014; 9: e96406.

6. Rosmalen JGM, Bos EH, De Jonge P. Validation of the Long-term Difficulties Inventory (LDI) and the List of Threatening Experiences (LTE) as measures of stress in epidemiological population-based cohort studies. *Psychol Med* 2012; 42: 2599–2608.

7. World Health Organization. Obesity and overweight, https://www.who.int/news-room/fact-sheets/detail/obesity-and-overweight. (accessed 10 July 2021).

8. Waist Circumference and Waist-Hip Ratio Report of a WHO Expert Consultation., http://apps.who.int/iris/bitstream/handle/10665/44583/9789241501491_eng.pdf;jsessionid=AE12D8579E6F3FBBE7BCFA69AF14560D?sequence=1 (2021).

9. Wendel-Vos GC, Schuit AJ, Saris WH, et al. Reproducibility and relative validity of the short questionnaire to assess health-enhancing physical activity. *J Clin Epidemiol* 2003; 56: 1163–1169.

10. Ainsworth BE, Haskell WL, Leon AS, et al. Compendium of physical activities: classification of energy costs of human physical activities. *Med Sci Sports Exerc* 1993; 25: 71–80.

11. Vinke PC, Corpeleijn E, Dekker LH, et al. Development of the food-based Lifelines Diet Score (LLDS) and its application in 129,369 Lifelines participants. *Eur J Clin Nutr* 2018; 72: 1111–1119.
